# P141: Media and hand hygiene: are healthcare workers receiving the correct example?

**DOI:** 10.1186/2047-2994-2-S1-P141

**Published:** 2013-06-20

**Authors:** E Spierings, M Nabuurs-Franssen, J Hopman, C Meijer, P Spierings, E Perencevich, A Voss

**Affiliations:** 1 Medical School, Radboud University Nijmegen, the Netherlands; 2 Department of Medical Microbiology and Infectious Diseases, Canisius-Wilhelmina Hospital, the Netherlands; 3 Department of Medical Microbiology, Radboud University Nijmegen, Nijmegen, the Netherlands; 4 Division of Infectious Diseases and Epidemiology, University of Iowa Hospital and Clinics, Iowa City, IA, USA

## Introduction

Attempts to increase hand hygiene compliance are complex and are - in parts - based on change of behavior. Especially the behavior of significant others (role models) was shown to be a strong motivator. While role models within the working environment are obviously the most important, some experts suggest that media and public display cannot be ignored.

Aim of the present study was to examine the display of “naked below the elbow”, which is considered a basic requirement for good hand hygiene in many countries, in sets of professional stock photos.

## Methods

From 20 random photo-stock websites we selected, twice 40 pictures with search term “doctor or nurse and patient”, respectively. In all selected photos a doctor or nurse and a patient were presented, HCWs were wearing white coats or uniforms, and the arms of the HCWs were visible.

Each photo was evaluated with regard to: closure of white coat, sleeve length, personal clothing covered, wrist watches & jewelry, and hairstyle.

## Results

Overall, 1600 photos were evaluated. The most common mistakes were with regard to HCW’s white coats/ uniforms. Overall, 39.9% of the pictures were correct with regard to all criteria evaluated; 68.6% of all those displaying nurses, and 11.3% of those displaying doctors.

## Conclusion

The results seem to reflect the real world with only 40% correct behavior and doctors being worse than nurses. It seems that the stereotype image of a doctor does not agree with the current hand hygiene guidelines. If we aim for higher compliance rates of HCWs, we need to change the social image of doctors.

## Disclosure of interest

None declared

